# LGR5 expression is regulated by EGF in early colorectal adenomas and governs EGFR inhibitor sensitivity

**DOI:** 10.1038/bjc.2017.412

**Published:** 2017-11-16

**Authors:** R G Morgan, E Mortensson, D N Legge, B Gupta, T J Collard, A Greenhough, A C Williams

**Affiliations:** 1 School of Cellular and Molecular Medicine, University of Bristol, Biomedical Sciences Building, University Walk, Bristol BS8 1TD, UK; 2 European Cancer Stem Cell Research Institute, Cardiff University, Hadyn Ellis Building, Maindy Road, Cardiff CF24 4HQ, UK

**Keywords:** LGR5, EGF, colorectal, adenoma, proliferation, survival, EGFRi, gefitinib

## Abstract

**Background:**

LGR5 serves as a co-receptor for Wnt/*β*-catenin signalling and marks normal intestinal stem cells; however, its role in colorectal cancer (CRC) remains controversial. LGR5^+^ cells are known to exist outside the stem cell niche during CRC progression, and the requirement for epidermal growth factor (EGF) signalling within early adenomas remains to be fully elucidated.

**Methods:**

Epidermal growth factor and gefitinib treatments were performed in EGF-responsive LGR5^+^ early adenoma RG/C2 cells. 2D growth assays were measured using an IncuCyte. LGR5 or MEK1/2 silencing studies were executed using siRNA and LGR5 expression was assessed by qRT–PCR and immunoblotting. Ki67 level and cell cycle status were analysed by flow cytometry.

**Results:**

Epidermal growth factor suppresses expression of LGR5 at both the transcript and protein level in colorectal adenoma and carcinoma cells. Suppression of LGR5 reduces the survival of EGF-treated adenoma cells by increasing detached cell yield but also inducing a proliferative state, as evidenced by elevated Ki67 level and enhanced cell cycle progression. Repression of LGR5 further increases the sensitivity of adenoma cells to EGFR inhibition.

**Conclusions:**

LGR5 has an important role in the EGF-mediated survival and proliferation of early adenoma cells and could have clinical utility in predicting response of CRC patients to EGFR therapy.

Colorectal cancer (CRC) is one of the most common cancers of the developed world (Ferlay *et al*, 2015) and progresses through a well-defined adenoma-carcinoma sequence (Fearon and Vogelstein, 1990). During malignant transformation, a tumour must adopt a variety of phenotypic states including inhibition of proliferation to promote survival, in order to respond and persist under various selection pressures (e.g., resource deprivation, immune predation or drug exposure) (Aktipis *et al*, 2013). Understanding how cancer cells are able to arrest growth in order to adapt to environmental insult is crucial in order to successfully treat CRC. Our previous work has shown that LGR5 (leucine-rich G-protein coupled receptor 5, Gpr49) serves as a pro-survival factor in early human colorectal adenoma cells (Al-Kharusi *et al*, 2013). LGR5 is a seven-transmembrane receptor protein that potentiates canonical Wnt signalling and is itself a downstream Wnt target gene, while also marking normal stem cells in multiple tissue types, including the colon (Barker *et al*, 2007). The advent of 3D organoid culture has shown that single isolated LGR5^+^ cells from the gut form self-organising crypt/villus structures that harbour the full repertoire of differentiated epithelial lineages of the intestine (Sato *et al*, 2009). Epidermal growth factor (EGF) is secreted by the Paneth cells in the niche to maintain LGR5^+^ stem cells (Sato *et al*, 2011b), and forms an essential component of organoid medium for the establishment of normal isolated LGR5^+^ stem cells in 3D culture (Sato *et al*, 2009). Studies examining the function of LGR5 in CRC cells have produced confusing and contradictory results, with some studies suggestive of a pro-tumourigenic role for LGR5 (Hirsch *et al*, 2014; Liu *et al*, 2014; Lin *et al*, 2015) while others indicate a tumour-suppressive role (de Sousa *et al*, 2011; Walker *et al*, 2011; Wu *et al*, 2014). Regardless of the ‘top-down’ or ‘bottom-up’ theories that have been proposed for CRC initiation (Shih *et al*, 2001; Preston *et al*, 2003), colorectal adenomas are known to harbour LGR5^+^ cells (Baker *et al*, 2015; Yanai *et al*, 2017). Furthermore, in mouse intestinal adenomas, it has been shown that LGR5^+^ cells are intermingled with Paneth cells in architecture reminiscent of the normal crypt which indicates LGR5^+^ cells remain dependent on secreted factors during early tumourigenesis (Schepers *et al*, 2012). The specific requirements for EGF signalling at this stage are not fully understood. EGF signalling is a critical pathway for normal intestinal homeostasis, and amplification or mutations to the EGF receptor (EGFR) or downstream signalling components such as KRAS are frequent in CRC and render promising EGFR therapies ineffective (Moroni *et al*, 2005; Lievre *et al*, 2006; van Houdt *et al*, 2010; Misale *et al*, 2012). However, there remains considerable variability in the clinical response of CRC patients to EGFR inhibition (EGFRi) despite wild-type *KRAS* or *EGFR* status, meaning additional factors can dictate EGFRi sensitivity (Blanke, 2005; Jimeno *et al*, 2009; Shaib *et al*, 2013). Our study shows for the first time that EGF exposure represses LGR5 expression in human colorectal adenoma and tumour cells at both the protein and transcriptional level. Furthermore, LGR5 silencing in adenoma cells enhances the proliferative state of EGF-treated adenoma cells, as observed by induction of Ki67 expression, enhanced cell cycle progression and reduced survival. Such observations suggest the modulation of LGR5 expression is important for directing the proliferative/survival phenotypes necessary for adenoma cell adaptation to environmental pressures. Importantly, the knockdown of LGR5 increases the sensitivity of adenoma cells to the EGFR inhibitor gefitinib, suggesting low LGR5 expression could be used clinically to predict patients who may benefit from EGFR therapies.

## Materials and methods

### Cell culture and treatments

The colorectal adenoma cell line RG/C2 was maintained as previously described (Al-Kharusi *et al*, 2013). For 2D growth measurements, RG/C2 adenoma cells were seeded at 1.25 × 10^4^ cells per well of a 96-well plate and treated with vehicle control (water) or 5, 10, 25 and 50 ng ml^−1^ EGF (Peprotech, London, UK) for 72 h. Prior to all EGF or gefitinib exposures, RG/C2 cells were grown in reduced serum (10% FBS) for 16 h. For siRNA experiments, RG/C2 cells were seeded at 5 × 10^5^ per ml and supplemented with either 2.5 ng ml^−1^ EGF or 5 *μ*M gefitinib. For 3D culture 5 × 10^2^ RG/C2 cells were seeded into 50 μl matrigel (Becton Dickinson, Oxford, UK) per well of a 24-well plate and bathed in medium previously optimised for colonic organoids (Sato *et al*, 2011a).

### Imaging

Widefield imaging of 3D cultures was performed *in situ* using a Leica DMI6000 inverted epifluorescence microscope (Leica, Buckinghamshire, UK) with a Leica DFC365FX monochrome digital camera in conjunction with LAS-X (Leica) acquisition software version 1.1.0.12420. Multiple fields were captured in a Z-stack through the matrigel and a minimum of 50 adenoma spheroids measured at their widest diameter using LAS AF software v2.6.0 (Leica). Post-acquisition refinement was performed using Photoshop CS6 v13.0 (Adobe, Berkshire, UK). For 2D growth assays, live real-time IncuCyte ZOOM software analysis masks (Essen BioScience, Ltd., Welwyn Garden City, Hertfordshire, UK) were used to measure cell growth. Data shown are from six replicates (24 images per time-point).

### Immunoblotting

LGR5 and *α*-tubulin immunoblotting was performed as previously described (Al-Kharusi *et al*, 2013). Antibodies were also used to EGFR, pEGFR, pERK1/2, LRP6, Frizzled-5, Axin-2, Survivin, CyclinD1, MEK1/2 (Cell Signaling Technology, Danvers, MA, USA), active *β*-catenin (Millipore, Billerica, MA, USA), and c-MYC (Santa Cruz Biotechnology, Dallas, TX, USA). All immunoblots shown are representative of three independent experiments and *α*-tubulin was used to assess protein loading.

### qRT–PCR

LGR5 qRT–PCR was performed as previously described (Al-Kharusi *et al*, 2013).

### RNA interference

LGR5 knockdown was performed using smartpool siRNA (GE Dharmacon, Buckinghamshire, UK) as previously described (Al-Kharusi *et al*, 2013). MEK1 knockdown was performed using smartpool siRNAs containing the following four target sequences; 5′-GCACAUGGAUGGAGGUUCU-3′, 5′-GCAGAGAGAGCAGAUUUGA-3′, 5′-GAGCAGAUUUGAAGCAACU-3′ and 5′-CCAGAAAGCUAAUUCAUCU-3′ (GE Dharmacon). MEK2 knockdown was performed using smartpool siRNAs containing the following four target sequences; 5′-CAAAGACGAUGACUUCGAA-3′, 5′-GAUCAGCAUUUGCAUGGAA-3′, 5′-GGAAGCUGAUCCACCUUGA-3′ and 5′-GAAAGUCAGCAUCGCGGUU-3′ (GE Dharmacon).

### Flow cytometric assessment of Ki67 and DNA content

For flow cytometric analyses, 5 × 10^5^ RG/C2 cells were trypsinised into single cell suspensions, washed in staining buffer (1 × PBS with 0.5% BSA) and fixed in 70% ice-cold ethanol. Following washing, cells were incubated with PE-conjugated Ki67 antibody, or the relevant isotype and concentration matched isotype control (BD) for 30 min. Following incubation, cells were washed and finally resuspended in staining buffer containing 2 *μ*l ml^−1^ DRAQ5 (Ebioscience, Altrincham, UK). Flow cytometric measurements were acquired and analysed as previously described (Morgan *et al*, 2015).

### Statistics

All statistical analyses were performed using GraphPad Prism v7.0 (GraphPad Software, Inc., San Diego, CA, USA). Significance of difference was assessed using a one-sample or students *t*-test with significance defined at *P*<0.05. Unless otherwise stated all data represent mean±1 standard deviation, *n*=3. Statistical significance is denoted by *P<0.05, **P<0.01, ***P<0.001 or n.s.=not significant.

## Results

### LGR5 is suppressed in EGF-responsive RG/C2 adenoma cells

**Figure 1:**
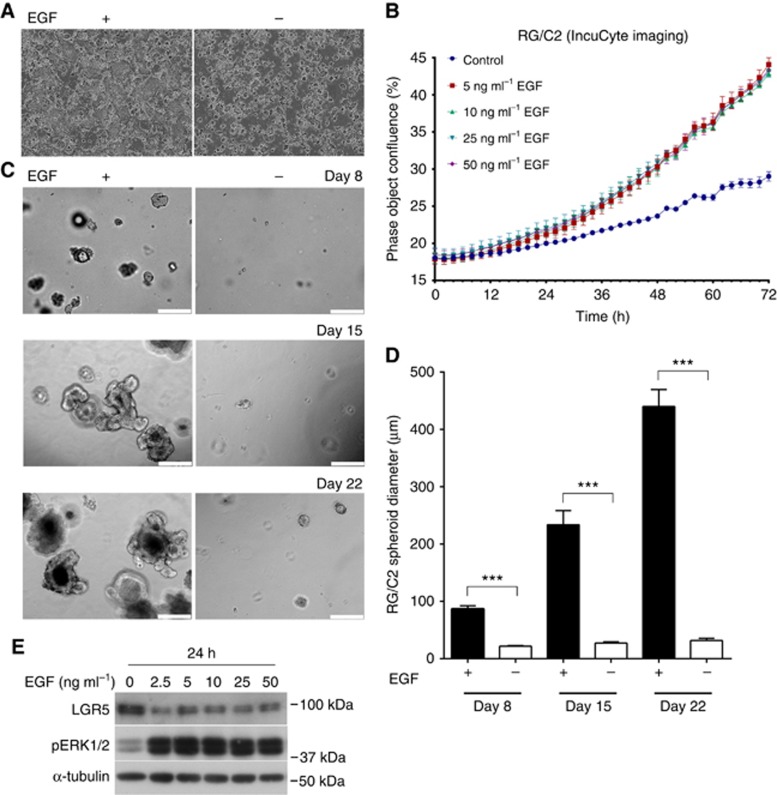
**LGR5 is suppressed in EGF-responsive RG/C2 adenoma cells.** (**A**) Representative IncuCyte images showing confluence of RG/C2 2D cell culture at 48 h +/− EGF (50 ng ml^−1^). (**B**) Summary graph showing RG/C2 cell 2D growth rate over 48 h +/− EGF. (**C**) Representative widefield microscopy images of RG/C2 adenoma spheroid morphology at 8, 15 and 22 days post-seeding following culture in 3D +/− EGF (50 ng ml^−1^). White scale bar indicates 250 μm. (**D**) Summary of average RG/C2 adenoma spheroid diameter following 8, 15 and 22 days post-seeding in 3D culture +/− EGF (50 ng ml^−1^). (**E**) Immunoblot showing expression of pERK1/2 and LGR5 protein in RG/C2 adenoma cells with a dose-response of EGF treatment. Statistical significance is denoted by ***P<0.001.

To assess the effect of EGF on LGR5^+^ cells at an early stage of CRC we required a physiologically relevant *in vitro* adenoma model that exhibited EGF-sensitivity. For this we utilised the RG/C2 adenoma cell line (*KRAS*^wt^) which harbours high levels of LGR5. Figure 1A and B demonstrate that the growth rate of RG/C2 adenoma cells in 2D is markedly enhanced in the presence of EGF over 72 h. Similar to normal primary murine intestinal LGR5^+^ cells (Sato *et al*, 2009), Figure 1C and D show RG/C2 adenoma cells are highly dependent on EGF supplementation for establishment and growth in 3D culture. In addition, RG/C2 adenoma spheroids behave similarly to LGR5^+^ cells in 3D culture, in that they exhibit enhanced differentiation-like features (branched/budding morphology) upon removal of the p38 MAPK inhibitor SB202190 from the medium (Supplementary Figure S1) (Otsuka *et al*, 2010; Sato *et al*, 2011a). EGF is a potent mitogen with activity in pico- and nanomolar ranges (Krall *et al*, 2011). To ascertain the concentration of EGF required for EGFR pathway activation in RG/C2 adenoma cells, we performed an EGF dose response over 24 h (Figure 1E). We observed that EGF treatment down to 2.5 ng ml^−1^ was sufficient to activate EGFR signalling in these cells as evidenced by optimal pERK1/2 induction. Perhaps unexpectedly, given the documented dependence of LGR5^+^ cells on EGF, we observed that EGF treatment suppressed LGR5 protein expression in adenoma cells at all EGF concentrations tested.

### EGF represses both LGR5 protein and mRNA expression

**Figure 2:**
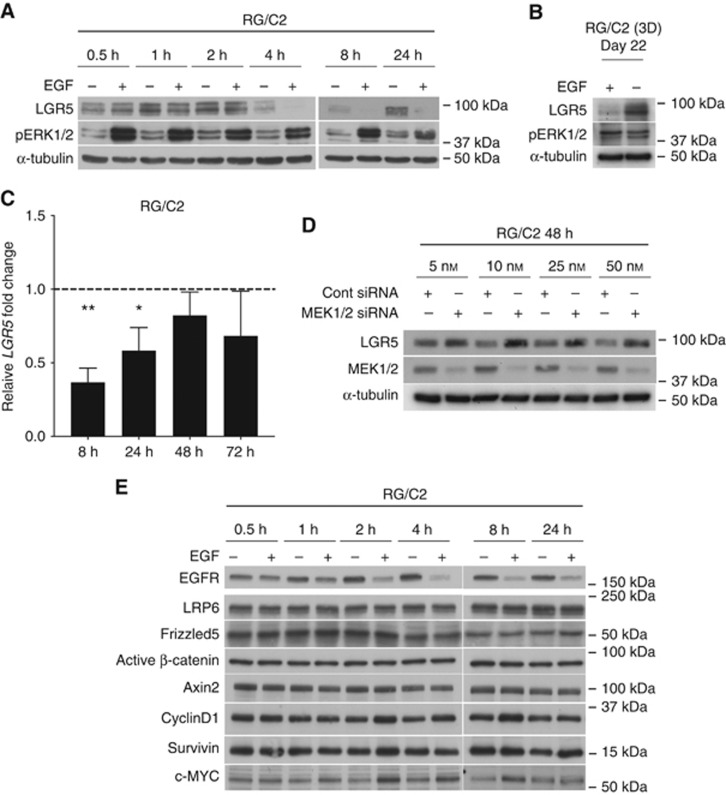
**EGF represses LGR5 expression, partly through MEK1/2.** (**A**) Representative immunoblot showing expression of LGR5 and pERK1/2 proteins in response to EGF time-course treatment (2.5 ng ml^−1^) of RG/C2 adenoma cells. (**B**) Representative immunoblot demonstrating LGR5 and pERK1/2 protein expression 24 h following EGF (50 ng ml^−1^) withdrawal from RG/C2 cells cultured in 3D for 22 days. (**C**) Summary of relative *LGR5* mRNA level in RG/C2 adenoma cells following 8, 24, 48 and 72 h EGF treatment. (**D**) Representative immunoblots showing LGR5 protein expression in response to 48 h treatment with dose-response of combined MEK1 and MEK2 siRNA. (**E**) Representative immunoblots showing expression of various Wnt components and target genes in response to EGF time-course treatment (2.5 ng ml^−1^) of RG/C2 adenoma cells (in samples from A). Statistical significance is denoted by *P<0.05, **P<0.01.

Given that we have previously demonstrated a short half-life for the LGR5 protein (Morgan *et al*, 2015), we further characterised the timing of LGR5 protein regulation by EGF in RG/C2 cells at multiple early time-points by immunoblotting. Figure 2A shows marked LGR5 suppression occurring as early as 4 h of EGF exposure. To assess LGR5 regulation by EGF within 3D adenoma culture, EGF was withdrawn for 24 h following 21 days culture in standard EGF/Noggin/R-Spondin (ENR) medium (since RG/C2 cells are exquisitely dependent on EGF for establishment in 3D culture) and LGR5 protein level was determined by immunoblotting. As predicted from 2D observations, EGF withdrawal from established 3D culture led to increased LGR5 expression in RG/C2 spheroids (Figure 2B). To understand the mechanism through which EGF regulates LGR5, we analysed *LGR5* mRNA levels in RG/C2 cells following 8, 24, 48 and 72 h EGF treatment. Figure 2C demonstrates how *LGR5* mRNA is significantly repressed at 8 and 24 h EGF treatment, and remains suppressed at 48 and 72 h (although this did not reach statistical significance). We also observed EGF-mediated suppression of LGR5 protein and/or mRNA expression at these same time-points in two CRC cell lines, SW620 and LoVo (Supplementary Figure S2). Of note, this regulation was not as marked as observed in the EGF-responsive RG/C2 cell line perhaps due to the presence of *KRAS* mutations and dysregulation of EGF signalling in these cell lines.

### LGR5 is partly regulated through MEK/ERK signalling

Since EGF activates MEK/ERK signalling (Figure 1E) (Giambartolomei *et al*, 2001), we silenced the key signalling intermediary proteins MEK1 and MEK2 in combination and subsequently examined LGR5 expression. Figure 2D demonstrates efficient knockdown of MEK1/2 proteins at 48 h at all siRNA concentrations assessed. In agreement with EGF exposure (MEK1/2 activation) repressing LGR5 protein expression, we observed that silencing of MEK1/2 proteins caused a consistent increase in LGR5 protein expression. Given that LGR5 is a Wnt target gene (Barker *et al*, 2007), and crosstalk exists between EGFR and Wnt/*β*-catenin signalling pathways (Hu and Li, 2010), we examined whether EGF regulation of LGR5 was indirect through inhibition of Wnt signalling. EGF-treated RG/C2 samples from Figure 2A were assessed for expression of Wnt signalling protein and target genes by immunoblotting. Figure 2E shows no significant alteration in Wnt signalling components or target genes was observed during EGF treatment. These data suggest that EGF could negatively regulate LGR5 expression in human colorectal adenoma cells in part through activation of MEK/ERK signalling.

### LGR5 repression reduces survival and enhances proliferative state of EGF-treated adenoma cells

**Figure 3:**
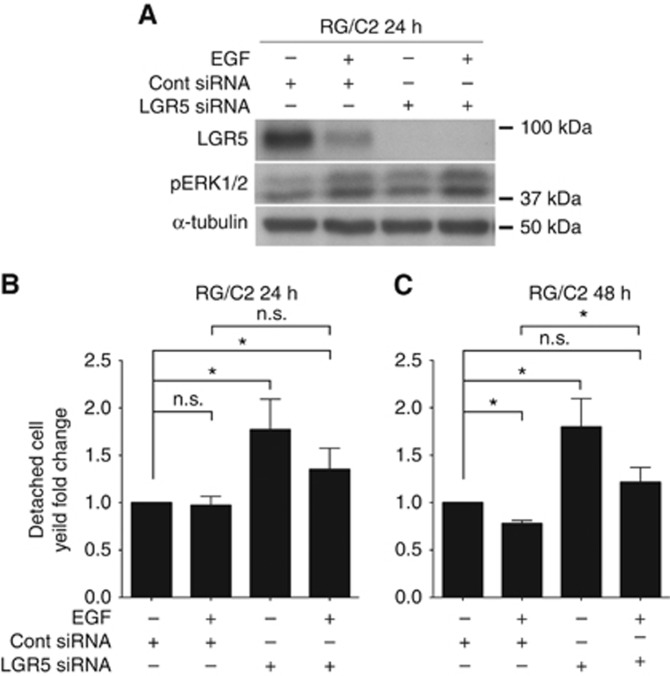
**LGR5 knockdown reduces the survival of EGF-treated adenoma cells.** (**A**) Representative immunoblots showing LGR5 and pERK1/2 protein level in response to both LGR5 siRNA +/− EGF treatment at 24 h. Summary graph of fold change in detached cell yield in response to LGR5 siRNA +/− EGF treatment at (**B**) 24 and (**C**) 48 h. Statistical significance is denoted by *P<0.05, or n.s.=not significant.

To understand the functional relevance of EGF-mediated suppression of LGR5, we adopted an LGR5 siRNA approach. Figure 3A shows similar LGR5 suppression upon EGF treatment (as previously observed in Figures 1 and 2), and confirms efficient knockdown of LGR5 protein with siRNA transfection at 24 h. To assess the impact of LGR5 knockdown on cell survival we measured detached floating adenoma cell yield as a measurement of cell death. Consistent with LGR5 being a pro-survival factor (Al-Kharusi *et al*, 2013), we observed a significant increase in detached cell yield at both 24 and 48 h following LGR5 siRNA transfection (Figure 3B and C). EGF treatment did not affect detached cell yield at 24 h but did result in a significantly lower number of apoptotic cells at 48 h as previously reported in the colon (Karnes *et al*, 1998; Suzuki *et al*, 2010). Finally, silencing of LGR5 increased the number of apoptotic cells present in EGF treated cultures at 24 h and significantly at 48 h.

**Figure 4:**
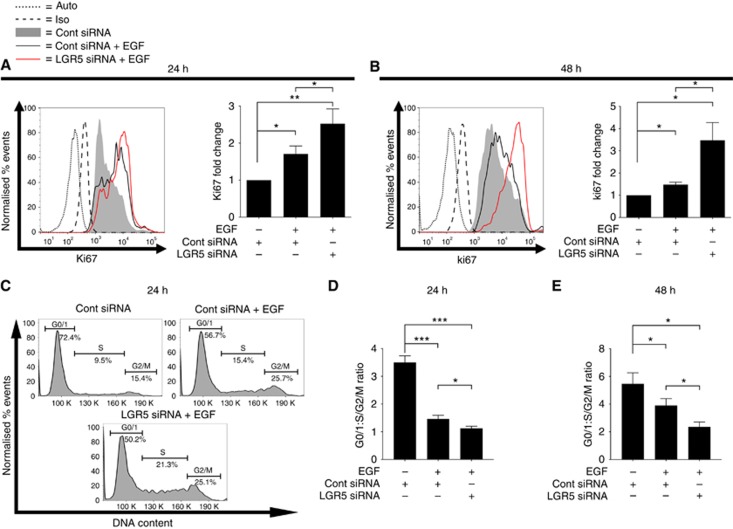
**LGR5 silencing enhances the proliferative state of EGF-treated adenoma cells through enhanced cell cycle progression.** Representative flow cytometric histograms, and summary graphs, showing internal levels of Ki67 proliferation marker in response to EGF treatment +/− LGR5 siRNA at (**A**) 24 and (**B**) 48 h. Auto=autofluoresence as determined from unstained cells, Iso=background fluorescence as determined from isotype control antibody stained cells. (**C**) Representative flow cytometric DNA histograms displaying cell cycle status of RG/C2 adenoma cells at 24 h EGF treatment +/− LGR5 siRNA. Graphs summarising the G0/1:S/G2/M ratio following EGF treatment +/− LGR5 siRNA at (**D**) 24 and (**E**) 48 h. Statistical significance is denoted by *P<0.05, **P<0.01, ***P<0.001.

To understand the basis for the reduced survival of EGF-treated adenoma cells upon LGR5 knockdown, we next examined expression levels of the Ki67 proliferation marker. EGF-treated adenoma cells exhibited a significantly higher level of Ki67 protein at both 24 and 48 h relative to control (Figure 4A and B) as expected given the growth response of RG/C2 cells to this mitogen (Figure 1A and B). Interestingly, at 24 and 48 h time-points, suppression of LGR5 expression in the EGF-treated cells significantly increased the level of Ki67 compared to control and EGF-treated cells alone. To understand the basis for the heightened proliferative state of EGF-treated adenoma cells upon LGR5 suppression, we next analysed cell cycle status. EGF-treated adenoma cultures contained a significantly higher proportion of cells in the S-phase or G2/M stages of the cell cycle, relative to control siRNA-treated cultures at both 24 and 48 h (Figure 4C–E). In keeping with Ki67 measurements, the combination of EGF and LGR5 siRNA significantly increased the proportion of cells in S-phase or G2/M stages of the cell cycle compared with the control or EGF alone, at both 24 and 48 h. Taken together these data indicate that suppression of LGR5 enhances the proliferative capacity of EGF-treated adenoma cells.

### LGR5 repression increases the sensitivity of adenoma cells to EGFR inhibition

**Figure 5:**
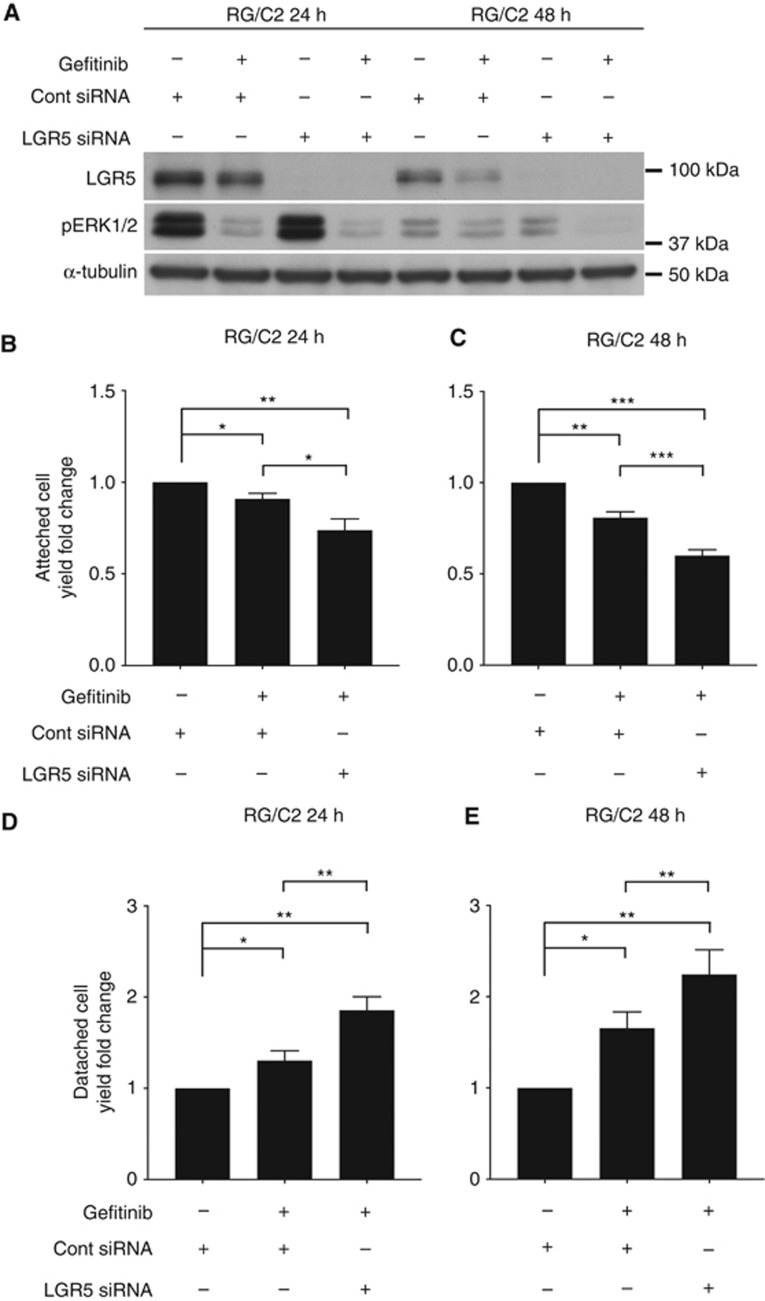
**Suppression of LGR5 enhances the sensitivity of adenoma cells to EGFR inhibition.** (**A**) Representative immunoblots showing LGR5 and pERK1/2 protein level in response to LGR5 siRNA +/− EGFRi treatment at 24 and 48 h. Summary graph of fold change in attached cell yield in response to EGFRi +/− LGR5 siRNA at (**B**) 24 and (**C**) 48 h. Summary graph of fold change in detached cell yield in response to EGFRi +/− LGR5 siRNA at (**D**) 24 and (**E**) 48 h. Statistical significance is denoted by *P<0.05, **P<0.01, ***P<0.001.

Given the enhanced proliferative response to EGF with LGR5 silencing, we next investigated whether this could sensitise adenoma cells to EGFRi. To examine this, we used LGR5 siRNA in combination with the EGFR inhibitor gefitinib (trade name Iressa). Western blot confirmed efficient LGR5 protein knockdown at both 24 and 48 h, and gefitinib efficiency with reduced pERK1/2 levels (Figure 5A). As observed from Figure 5B, EGFRi resulted in a small but significant reduction in the attached cell yield over 24 h, which was significantly augmented by the suppression of LGR5. A similar result was obtained following 48 h exposure of gefitinib, albeit a higher degree of growth inhibition had occurred in the EGFRi cultures, and significantly more with the combination of EGFRi- and LGR5 RNAi-treated cultures (Figure 5C). Detached cell yield was higher in RG/C2 adenoma cells treated with EGFRi but this was significantly enhanced by combination with LGR5 siRNA at both 24 and 48 h (Figure 5D and E).

## Discussion

Epidermal growth factor is an essential mitogen for the growth, survival and development of the normal intestine. Detailed analyses of the crypt base have shown neighbouring Paneth cells secrete EGF to maintain the LGR5^+^ stem cell pool, while the advent of organoid culture has demonstrated EGF to be one of the essential components required for the survival and maintenance of intestinal stem cells *in vitro* (Sato *et al*, 2009, 2011b). A recent study by Basak *et al* reported that EGF withdrawal from the ENR medium abolished the proliferation of LGR5^+^ cells, induced quiescence (via reduced MEK signalling), and led to a twofold increase in *LGR5* expression in normal primary mouse organoids (Basak *et al*, 2017). Our study examined the dependence of early human colorectal adenoma cells to EGF and compliments the above report by showing that the dependence on EGF for the proliferation of LGR5+ cells is retained by adenomas. Similar to normal murine organoids, we noted an increase in LGR5 expression following EGF withdrawal from 3D adenoma culture medium (Figure 2B). We also found EGF-mediated suppression of LGR5 to occur through a MEK1/2-associated mechanism as supported by the findings of another study where induced BRAF mutations (MAPK activation) led to loss of *LGR5* expression and the intestinal stem cell pool (Riemer *et al*, 2015).

The majority of our study was performed in a single human adenoma cell line (RG/C2); however, given the technical challenge of obtaining early human adenoma tissue that has not yet accrued *RAS, RAF* or *EGFR* mutations (which affect EGF signalling), this model remains a valued resource for studying the early molecular changes that occur during colonic adenoma progression. To improve our understanding of the EGF–LGR5 axis for human colorectal adenoma progression, further studies could be performed in a primary organoid culture system where the stepwise accumulation of genetic mutations essential for colorectal transformation (e.g., *APC, KRAS, P53, SMAD4*) can be controlled (Drost *et al*, 2015; Matano *et al*, 2015). Nether-the-less, these results build on our previous identification of LGR5 as a pro-survival factor in PGE2-treated adenoma cells (Al-Kharusi *et al*, 2013) and indicate that lowering of LGR5 expression could be an important event for adenoma-carcinoma progression. Indeed, the majority of adenoma cell lines contain high LGR5 expression, while most carcinoma cell lines exhibit low or absent LGR5 (except for metastatic cell lines) (Al-Kharusi *et al*, 2013)). De Sousa *et al* showed in primary CRC tissue that loss of Wnt target gene expression such as *LGR5* was frequent during adenoma-carcinoma progression (de Sousa *et al*, 2011) and other studies have reported pro-proliferative states upon LGR5 reduction (Walker *et al*, 2011). This fits too with the normal physiology of the intestinal crypt since LGR5 expression is lost as stem cells exit the niche and generate rapidly cycling progeny within the transit amplifying zone (Barker *et al*, 2007). The re-emergence of LGR5 expression in metastatic cell lines has perhaps recently been explained by de Sousa and colleagues, where LGR5^+^ cells were dispensable for an already established colorectal tumour, but were required for re-establishment (and survival) of the tumour at a distant site (Melo *et al*, 2017).

Vitally, our studies have also indicated a novel role for LGR5 expression in the sensitivity of adenoma cells to EGFR inhibitors such as gefitinib. The proliferative response increased by LGR5 suppression potentiates EGFRi sensitivity and death of adenoma cells (in keeping with the proliferative capacity of aggressive tumours rendering them more sensitive to anticancer agents (Valeriote and van Putten, 1975)). Gefitinib has previously shown promise as a combinatorial agent to existing chemotherapeutic regimens for CRC (Kuo *et al*, 2005; Ogino *et al*, 2005; Fisher *et al*, 2008). However, a longstanding clinical challenge has been the heterogeneous response of CRC patients to EGFRi despite wild-type *EGFR* or *KRAS* status (Blanke, 2005; Jimeno *et al*, 2009; Shaib *et al*, 2013). This implies the contribution of additional factors to EGFRi sensitivity and this study suggests tumours with low LGR5 expression will exhibit increased sensitivity. Given that EGFR has recently been identified as a biomarker at the adenoma stage for more aggressive CRC progression (Williet *et al*, 2017), our findings suggest that there could also be clinical benefit in assessing LGR5 expression at this early stage in order to stratify those patients who may respond best to EGFR therapy. LGR5 inhibitors have not been reported, but our data would suggest a combinatorial approach with EGFRi may synergise to reduce the survival of CRC cells.
