# Supplementary material for: LGR5 expression is regulated by EGF in early colorectal adenomas and governs EGFR inhibitor sensitivity
Source: Br J Cancer. 2017 Nov 16;118(4):558–65. doi: 10.1038/bjc.2017.412 (PMC5830587; doi:10.1038/bjc.2017.412)
Supplement: Supplementary Figures [file bjc2017412x1.pdf]

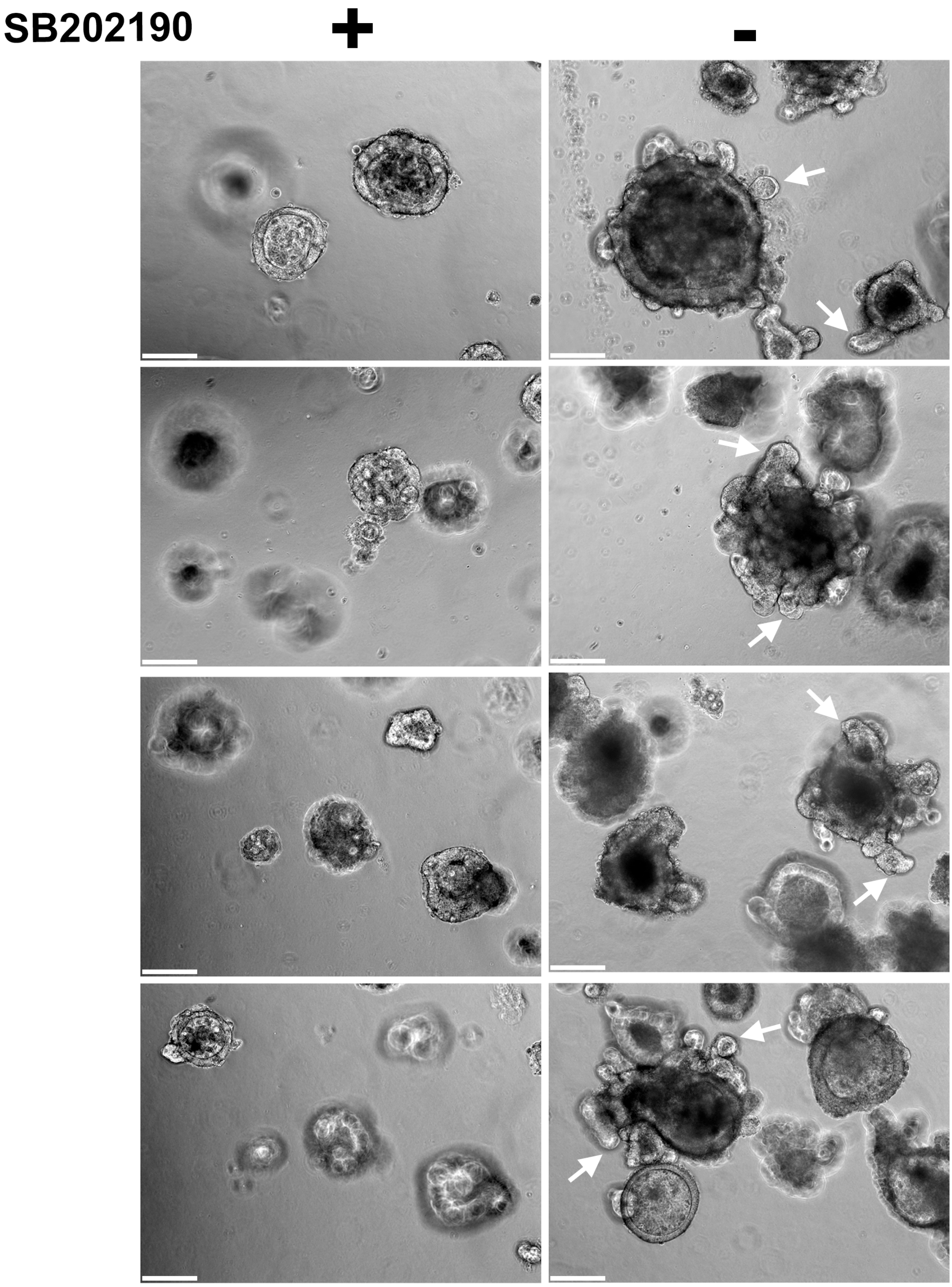

Supplemental Figure S1. RG/C2 adenoma spheroids behave similar to normal LGR5^{+} cells in 3D organoid culture. Representative widefield microscopy images (10x magnification) of RG/C2 adenoma spheroids in 3D culture (22 days post-seeding). Withdrawal of the p38 MAPK inhibitor SB202190 (10μM) results in emergence of budding/branching ‘differentiation-like’ features as exampled by white arrows. White scale bars = 190μM.

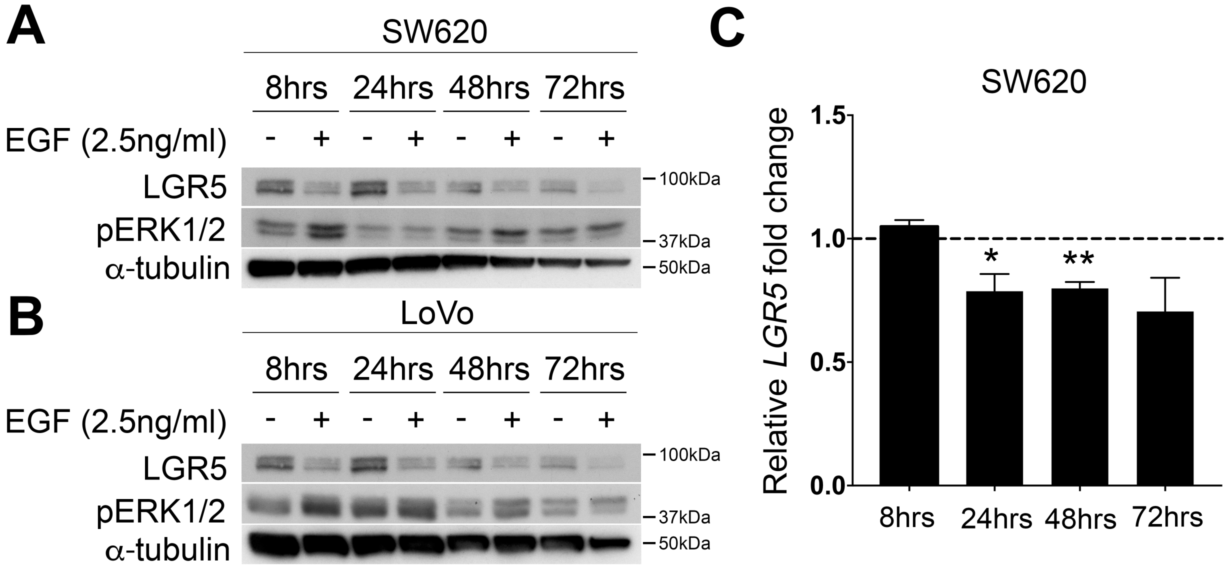

Supplemental Figure S2. EGF represses LGR5 protein and mRNA expression in metastatic colorectal carcinoma cell lines. Representative immunoblots showing expression of LGR5 and pERK1/2 proteins in response to EGF time-course treatment (2.5ng/ml) of (**A**) SW620 and (**B**) LoVo carcinoma cells (cultured as previously described (Al Kharusi *et al*, 2013). (**C**) Summary of relative *LGR5* mRNA level in SW620 carcinoma cells following 8, 24, 48 and 72 hours of EGF treatment. The loading control α-tubulin was used to assess relative loading between samples at matched time-points.
